# Capacity, Fidelity, and Noise Tolerance of Associative Spatial-Temporal Memories Based on Memristive Neuromorphic Networks

**DOI:** 10.3389/fnins.2018.00195

**Published:** 2018-03-28

**Authors:** Dmitri Gavrilov, Dmitri Strukov, Konstantin K. Likharev

**Affiliations:** ^1^Department of Electrical and Computer Engineering, Stony Brook University, Stony Brook, NY, United States; ^2^Department of Electrical and Computer Engineering, University of California, Santa Barbara, Santa Barbara, CA, United States; ^3^Department of Physics and Astronomy, Stony Brook University, Stony Brook, NY, United States

**Keywords:** spatial-temporal memories, associative memories, nanoelectronics, neuromorphic networks, memristors, CrossNets, capacity, noise tolerance

## Abstract

We have calculated key characteristics of associative (content-addressable) spatial-temporal memories based on neuromorphic networks with restricted connectivity—“CrossNets.” Such networks may be naturally implemented in nanoelectronic hardware using hybrid memristive circuits, which may feature extremely high energy efficiency, approaching that of biological cortical circuits, at much higher operation speed. Our numerical simulations, in some cases confirmed by analytical calculations, show that the characteristics depend substantially on the method of information recording into the memory. Of the four methods we have explored, two methods look especially promising—one based on the quadratic programming, and the other one being a specific discrete version of the gradient descent. The latter method provides a slightly lower memory capacity (at the same fidelity) then the former one, but it allows local recording, which may be more readily implemented in nanoelectronic hardware. Most importantly, at the synchronous retrieval, both methods provide a capacity higher than that of the well-known Ternary Content-Addressable Memories with the same number of nonvolatile memory cells (e.g., memristors), though the input noise immunity of the CrossNet memories is lower.

## Introduction

Associative spatial-temporal memories (ASTM), which record a time sequence of similarly-formatted spatial patterns, and then may reproduce the whole sequence upon the input of just one of these patterns (possibly, contaminated by noise), are valuable parts of cognitive systems. Indeed, we all know how a few overheard notes trigger our memory of an almost-forgotten tune. Such observations have been confirmed by detailed neurobiological studies of “episodic memories”, apparently localized in the hippocampus—see, e.g., the recent review by Eichenbaum (2013). Another example (which also gives a very natural language for the description of spatial-temporal patterns, used in this paper), is a reproduction of a movie, triggered by the input of its one, possibly incomplete or partly corrupted, frame. More generally, multi-dimensional associative memories may be used for a broad range of cognitive tasks—see, e.g., Imani et al. (2017) for recent literature.

The recent fast progress of mixed-signal nanoelectronic hardware, in particular of hybrid memristive circuits. Such circuits, which are based on nanoelectronic crossbars, with a memristive device (for example, a metal-oxide memristor) at each crosspoint (see e.g., the reviews by Likharev, 2008; Yang et al., 2013), may enable ASTMs with extremely high speed and energy efficiency. One option here is to use the so-called Ternary Content-Addressable Memory (T-CAM) architecture—(see, e.g., Pagiamtzis and Sheikholeslami, 2006). Indeed, as was discussed by Alibart et al. (2011), the memristive version of such a memory requires just two crosspoint devices per cell. As a result, the total number *n* of such devices in an associative memory holding *Q* spatial patterns (“frames”), of *N* bits (“binary pixels”) each, is just *2NQ*, i.e., is only twice larger than that necessary for the usual binary resistive memory, with no noise correction ability (It will be more convenient for us to discuss it operation in section Comparison with T-CAM).

**Figure 1:**
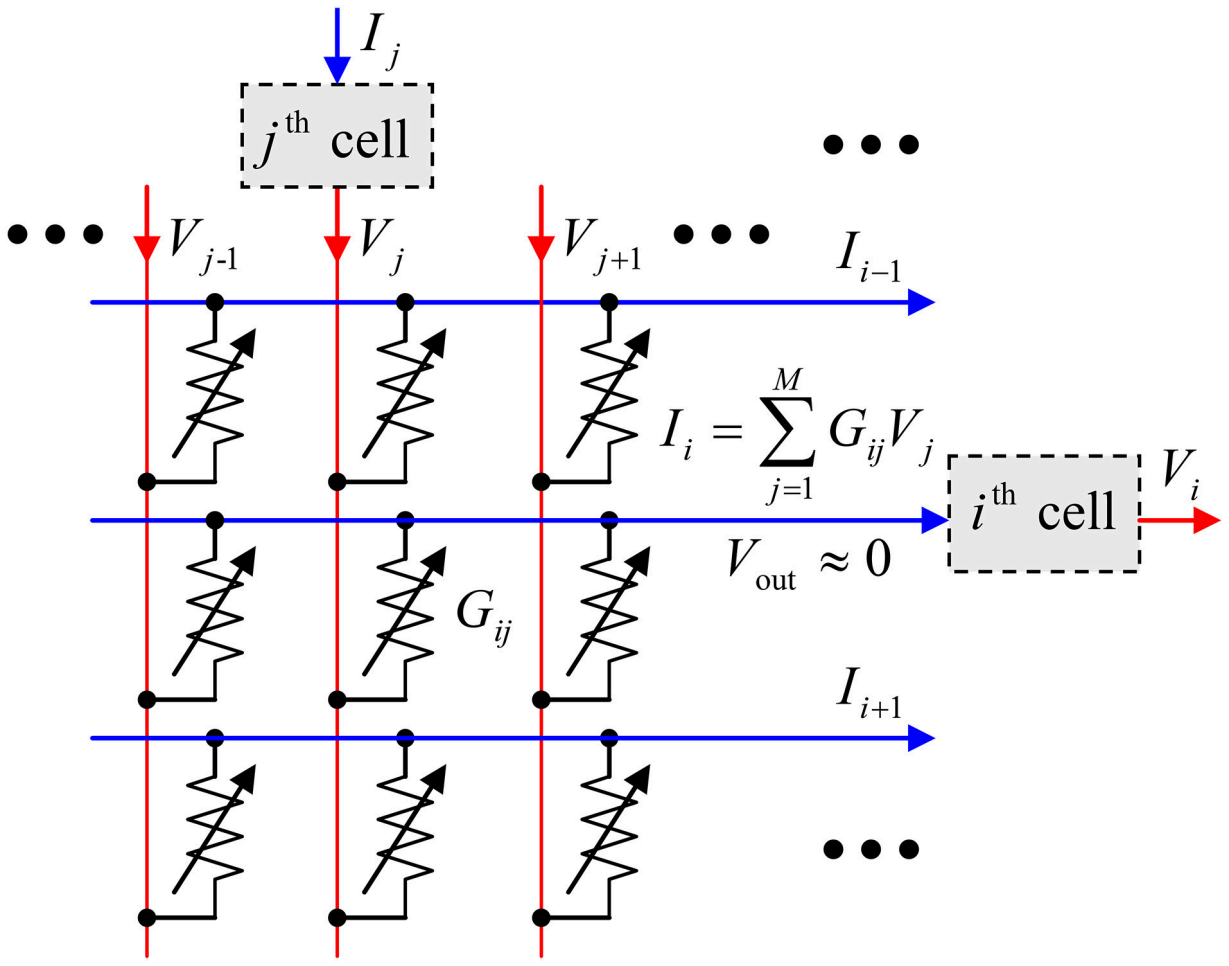
The equivalent circuit of the simplest memristive crossbar that can provide adjustable, nonvolatile coupling between neural cells.

In this paper, we will show that these hardware costs may be reduced even further using the hybrid neuromorphic networks with “CrossNets” architecture (see, e.g., Fölling et al., 2001; Türel et al., 2004; Likharev, 2011; Merrikh-Bayat et al., 2015; Adam et al., 2017), in which continuous-state memristive synaptic devices work together with CMOS-implemented neural cells—see Figure 1. If the voltages *V*_*j*_, developed by the neural cells and applied to the crossbar input lines, are not too large (for typical metal-oxide memristors, below ~1 V), they do not alter the pre-set states of the crosspoint devices, and the crossbar, with the virtual-ground condition *V*_out_ ≈ 0 enforced on its output lines, performs a multiplication of the vector of these voltages by the matrix of synaptic weights:

(1) $$\begin{array}{r} I_{i}=\overset{M}{\underset{j\;=\;1}{\sum }}w_{ij}V_{j}, \end{array}$$

where *I*_*i*_ is the output current (which serves as an input signal for the recipient neural cell), *M* is the cell connectivity, and the synaptic weight *w*_*ij*_ is, in the simplest case, proportional to the Ohmic conductance *G*_*ij*_ of the corresponding device (A modification of the relation *w*_*ij*_ ∞ *G*_*ij*_, beneficial for practical implementation, will be discussed in section Readout Options). Hence the memristive crossbar, with continually and precisely adjustable crosspoint devices, can perform, on the physical level, the neuromorphic network's most common inference-stage operation, which is the main bottleneck at their digital implementation. As a result, the intercell communication delays in nanoelectronic CrossNets may be reduced to just few nanoseconds, and their energy efficiency may approach that of the human cerebral cortex.

**Figure 2:**
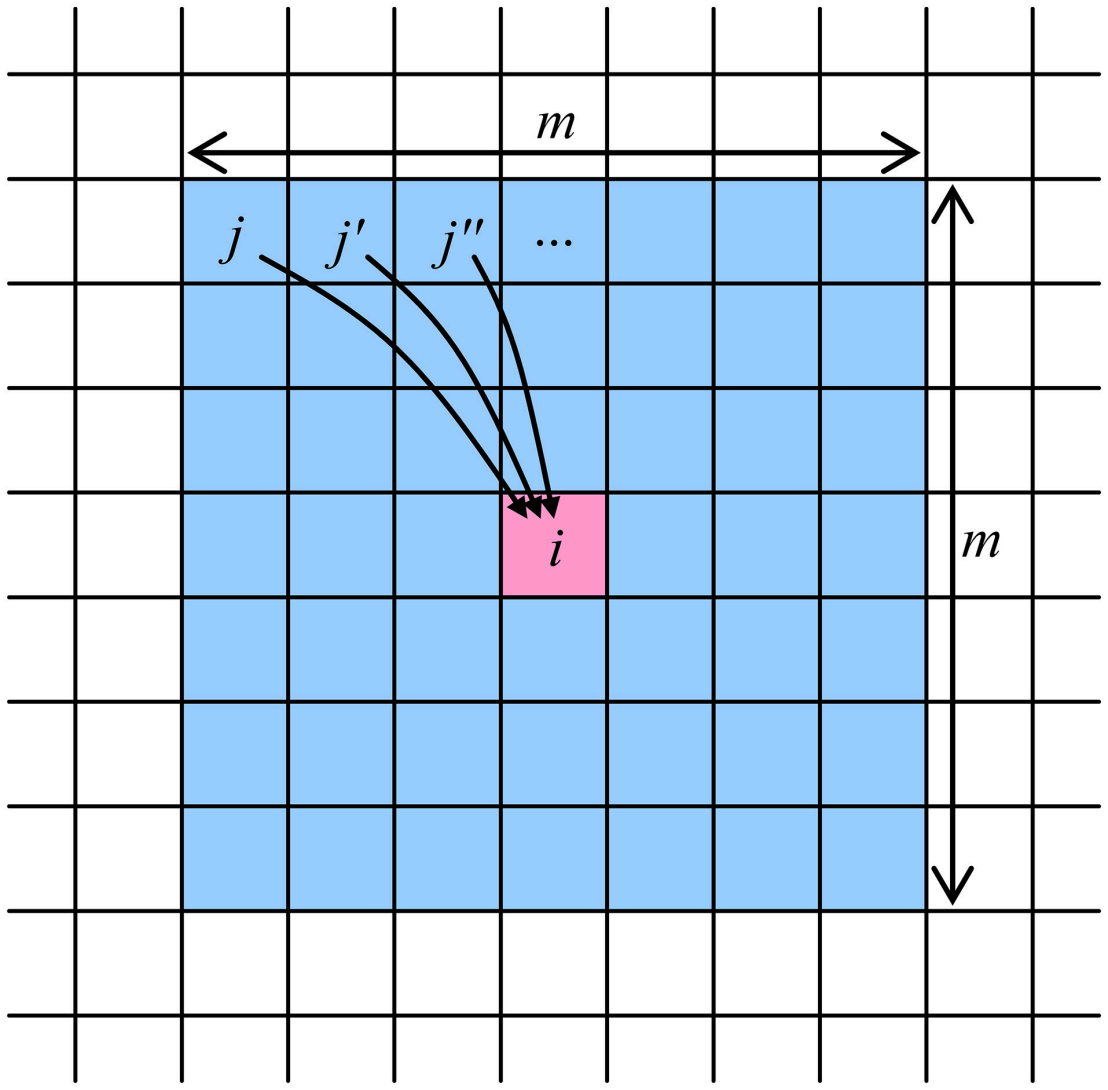
The connectivity domain of a neuron cell number *i*, which may be provided by a memristive CrossNet with the InBar topology (Türel et al., 2004). Note that each cell has a similar domain, so that the connection between the cells of each pair is two-sided—though typically asymmetric.

The global connectivity of a limited number *N* of neuron cells, with *M* = *N* – 1, may be implemented by placing the cells peripherally, around a single *N* × *N* crossbar. However, for most real-world applications, such global connectivity is redundant, and an area-distributed interface between a memristive crossbar and an array of CMOS-implemented neurons may be used to provide the desired restricted connectivity graph. For example, the very natural “InBar” interface topology (Türel et al., 2004) may ensure the connectivity of each neuron with all other neurons in its vicinity with a shape approaching that of a square *m* × *m*, so that *M* = *m*^2^ – 1 < *N*, see Figure 2 (For practically interesting cases, 1 < < *M* < < *N*). Such shape of the connectivity domain is very convenient for the discussion of the CrossNet ASTM (though not necessary for its physical implementation), and will be used in this paper.

**Figure 3:**
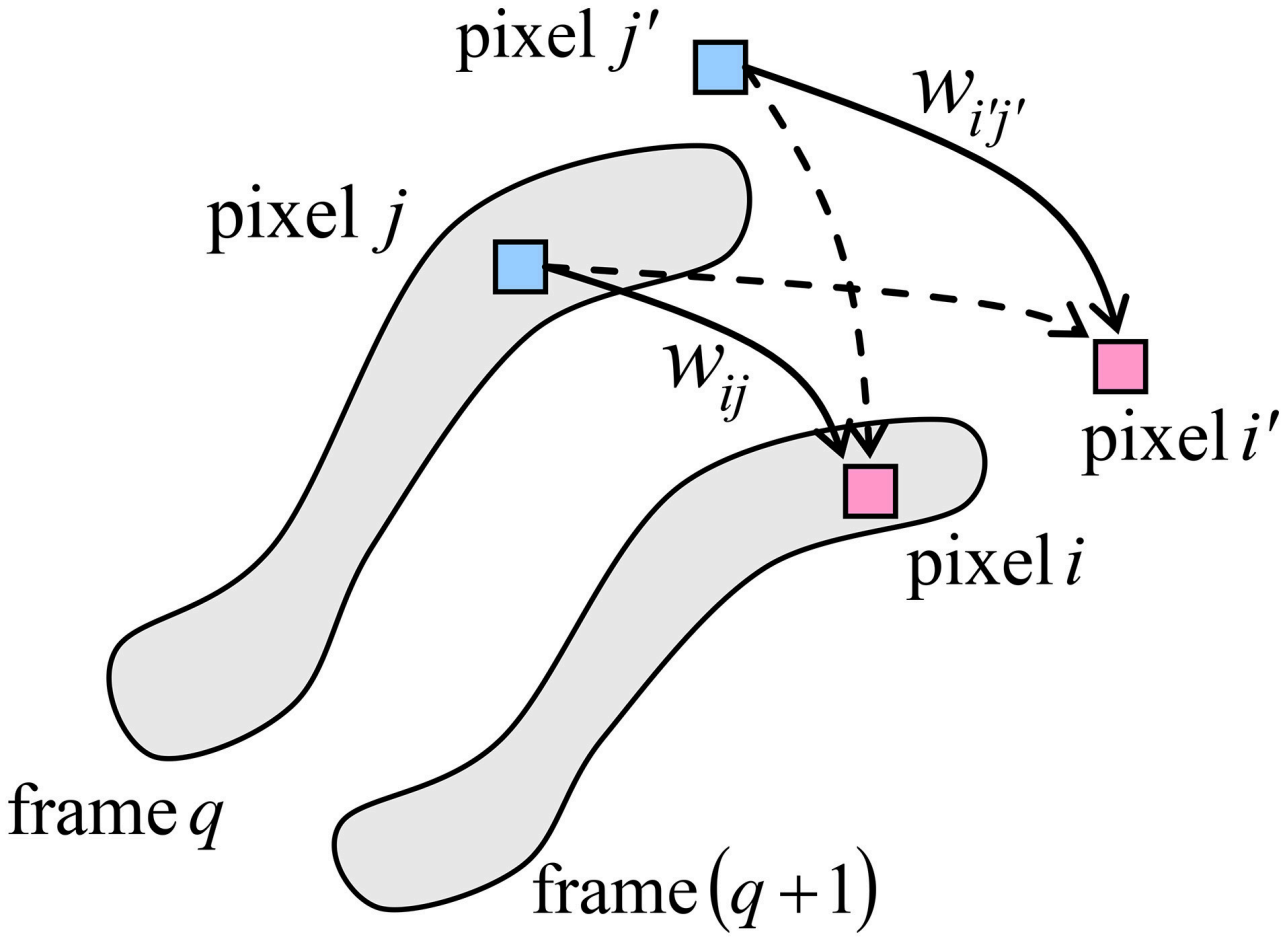
The basic idea of operation of the neuromorphic ASTM. The shaded areas show the set of pixels “active” (say, black) in each of the two sequential frames.

Figure 3 shows the basic idea of operation of the memory. Just as in Figure 2, the neural cells are mapped on a rectangular grid, each cell corresponding to one B/W pixel of all movie frames. At the movie recording stage, for each pair of sequential frames, the synaptic weight connecting two pixels, within their connectivity domain, is strengthened if the two pixels have the same value (1 or 0) in both frames, and is weakened in the opposite case. For example, in the case of Figure 3, where the pixels of a certain value (say, 1) are placed on gray background, the weights *w*_*ij*_ and $w_{i^{\prime }j^{\prime }}$ (symbolized by solid lines) are strengthened, while the weights $w_{i^{\prime }j}$ and $w_{ij^{\prime }}$ (dashed lines) are weakened.

**Figure 4:**
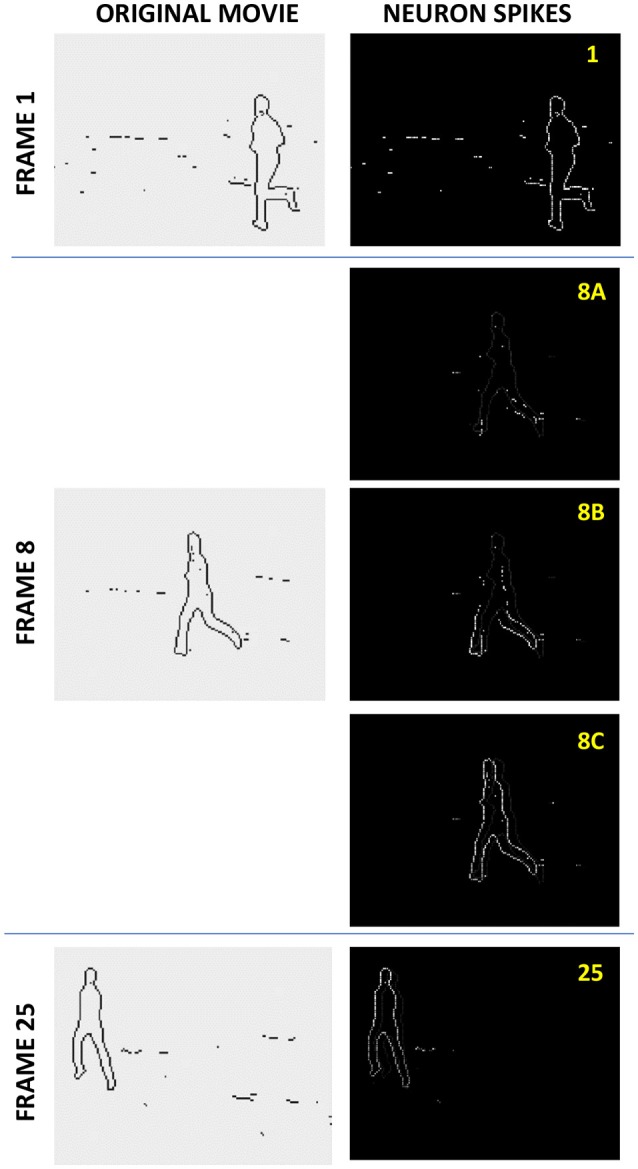
The spike jitter effect at the asynchronous memory retrieval. A set of 4 different movies, with 25 frames each, each consisting of *N* = 120 × 160 B/W pixels (obtained by applying edge detection to grayscale movies showing running humans), was recorded into a simulated ASTM of the same size (120 × 160 LIF cells), with connectivity *M* = 31 × 31 – 1. The left column shows three frames of one of the original movies: the initial Frame 1, an intermediate Frame 8, and the final Frame 25. The right column shows the brightness-coded snapshots of the spikes at the retrieval of this movie, with three middle snapshots, corresponding to Frame 8, separated by very small time intervals. As discussed in section Readout Options, the figure demonstrates the (rather counter-intuitive) effect of non-accumulating spike jitter.

If the recording procedure has been efficient, then at the readout (also called the “retrieval”) stage, the activation of pixels by those of an input frame leads to a correct sequential activation of the following frames of the movie, even if the input frame is either incomplete, or partly corrupted by noise. Figure 4 shows an example of such operation. A set of 4 different movies, with 25 frames each, obtained by applying edge detection to grayscale movies showing running humans, was recorded into a simulated ASTM of the same size (*N* = 120 × 160 = 19,200), with connectivity *M* = 31 × 31 – 1 = 960. The left column shows three frames of one of the original movies: the initial Frame 1, an intermediate Frame 8, and the final Frame 25. The right column shows the brightness-coded snapshots of the spikes at the retrieval, triggered by the first frame of this particular movie (Three middle snapshots correspond to the same recorded Frame 8, separated by very small time intervals; their difference will be explained in section Readout Options below).

One can see that the movie retrieval is almost perfect (The same network, without any change of synaptic weights *w*_*ij*_, gives an equally fair reproduction of any of 3 other movies, when triggered by its frame). However, such faithful retrieval is only possible when the total number *Q* of the frames does not exceed a certain number *Q*_max_, called the memory capacity. This limitation, *Q* < *Q*_max_ is due to the fact that different frame pairs typically impose contradictory requirements on the same synaptic weight *w*_*ij*_.

The general idea of such operation of the ASTM is not quite new. Its software aspects were repeatedly discussed starting from the 1960s—see, e.g., Grossberg (1969). A review of the initial work, mostly for the firing-rate networks, may be found in section 3.5 of Hertz et al. (1991). This idea was revitalized (Gerstner et al., 1993) at the advent of spiking network research, and in this context, discussed in quite a few publications—see, e.g., the reviews (Kremer, 2001; Wörgötter and Porr, 2005), and later papers (Yoshioka et al., 2007; Brea et al., 2011; Nguen et al., 2012; Kabasov et al., 2013; Yu et al., 2016). However, to the best of our knowledge, the key issue of the ASTM capacity was addressed only in the Ph.D. thesis by Wills (2004), for a very specific readout timing model, very inconvenient for hardware implementation (The capacity calculated in that work is also substantially lower than for the best readout methods described below).

The objective of this work was a detailed study of the recording and readout methods, which would enable the highest capacity of the CrossNet ASTM. In the next section, we start with the discussion of the readout options, in particular the most critical issue of readout timing, and proceed to the definition of four most plausible ways of data recording. The following section contains the results of analysis of the proposed methods, including calculation of the corresponding capacity-vs.-fidelity tradeoffs. The best two recording methods are discussed in more detail, with emphasis on their immunity to the input frame corruption and crosspoint device variability. The section ends with the comparison of the performance of ASTM with that of the memristive T-CAM suggested by Alibart et al. (2011). Finally, in the Discussion section we summarize our results, and discuss prospects of experimental implementation of ultrafast CrossNet ASTM.

## Methods for recording and retrieving data for ASTM

### Readout options

As follows from the above qualitative description of the memory, it is quite suitable for the asynchronous spiking mode of operation—see, e.g., Gerstner and Kistler (2002). In this mode, the input of all initial frame's “active” pixels (say, equal to 1) triggers simultaneous spikes *V*_*j*_(*t*) at the outputs of the corresponding neural cells. As a result of their action on the memristive crossbar, all other cells of the system receive input pulses *I*_*i*_(*t*) described by Equation (1). In some of the cells (ideally, all and only those corresponding to the active pixels of the next frame), the input pulses promote the action potential beyond the spiking threshold, causing them to fire. This new series of spikes triggers spiking in the next cell set, corresponding to the active pixels of next frame, etc.

We have carried out extensive numerical experiments with this readout mode, using the simple leaky integrate-and-fire (LIF) model of the cells (Gerstner and Kistler, 2002), and the following shape of the spike:

$$\begin{array}{l} g\left(t\right)=C\sin\left(\frac{t}{\tau }\right)\exp\left(-\frac{t}{2\tau }\right),t\geq 0, \end{array}$$

where constant *C* is used for scaling the amplitude of the spike and time constant τ is selected based on the desired spike duration. The particular spike shape and the values of C and τ are not critical for the effects discussed in this paper.

The simulations have shown the following very interesting behavior, illustrated by Figure 4. In the absence of global synchronization, the cells corresponding to active pixels of a frame (besides the initial one) do not necessarily fire simultaneously, because of the previously accumulated individual action potentials, which are practically random. Only the initial frame 1, triggered by the simultaneous input spikes, is reproduced perfectly. In the typical intermediate frame 8, the spikes are spread in time—see the readout snapshots 8A−8C, made with small time intervals between them. If the number *Q* of the recorded frames is much smaller than the memory capacity *Q*_max_ (for a particular recording method), this “jitter” of the spikes is almost negligible. As *Q* is increased, the jitter also increases, but the spikes belonging to each frame remain clustered in time, with the cluster width not exceeding the average distance between the frames, and time-averaged contents of each frame is still reproduced correctly, i.e., the jitter does not accumulate—see, e.g., the much later frame 25 in Figure 4. Only when *Q* approaches *Q*_max_, the spiking time clusters are getting blurred, and the reproduced movie eventually degrades into noise.

We believe that the observed effect may be rather interesting for theoretical neuroscience, and has to be studied in more detail. However, we could not help noticing that the elementary global timing (synchronization) of all spikes of each frame kills this jitter, simultaneously increasing the memory capacity *Q*_max_ rather dramatically. Such global timing may be achieved without much hardware overhead—for example, just by a periodic simultaneous lowering of the firing thresholds of all the cells, with a time period somewhat larger than the characteristic time of the *RC*-transient in the crossbar.

Because of this, the qualitative results presented in the balance of this paper are for the globally-synchronous readout mode. In order to analyze this mode, we have used the following simple model (which blurs the difference between spiking and firing-rate operation): for each time period, corresponding to the reproduction of one frame of the movie, the voltages *V*_*j*_ and currents *I*_*i*_ in Equation (1) are considered constant, with each neural cell providing a static threshold activation function *V*_*i*_^(q+1)^ = *f* (*I*_*i*_^(q)^), where the upper index is the frame number (*q* = 1, 2, …, *Q*). The activation function was taken in the simple form

(2) $$\begin{array}{r} V_{i}^{\left(q+1\right)}=V_{0}\text{ sgn }I_{i}^{\left(q\right)}, \end{array}$$

where *V*_0_ is a constant coefficient, selected for convenience of implementation, and influencing only the scaling of synaptic weights (In our simulations, we set *V*_0_ = 1 without loss of result generality). The relation (2) implies the zero-centered operation mode, in which *V*_*i*_, *I*_*i*_, and *w*_*ij*_ may be either positive or negative. This mode may be naturally implemented in differential CrossNets, in which the *j*th cell contribution to the current input of the *i*th cell is the sum of currents through two crosspoint devices (both with positive conductances *G*), fed by equal voltages of opposite polarities:

$$\begin{array}{l} I_{i}=\overset{M}{\underset{j\;=\;1}{\sum }}\left(G_{ij}^{+}V_{j}-G_{ij}^{-}V_{j}\right) \end{array}$$

so that the effective synaptic weight *w*_*ij*_ ∞ *G*_*ij*_^+^ – *G*_*ij*_^−^ may have an arbitrary sign (Türel et al., 2004); such mode is also convenient for the compensation of the temperature dependence of memristor conductances—see, e.g., Prezioso et al. (2015).

### Recording methods

At the first stage of our work, we have explored the tradeoff between the movie retrieval fidelity (in terms of the probability of the correct readout, in a statistical ensemble of random frames) and the network capacity *Q*_max_, for four most natural methods of movie recording, temporarily assuming perfect hardware operation.

#### The Hebb rule

Conceptually, the most straightforward recording method is using the Hebb rule in its simplest form (Amari, 1972):

(3) $$\begin{array}{r} w_{ij}=\frac{1}{Q}\overset{Q}{\underset{q\;=\;1}{\sum }}s_{i}^{\left(q+1\right)}s_{j}^{\left(q\right)}, \end{array}$$

where *s*_*j*_^(*q*)^ = ±1 are the symmetrized values of the B/W pixels of the *q*th frame. This rule evidently corresponds to the verbal description of the weight setup discussed in the Introduction. It is suitable for *in situ* recording in hardware, using the spike-time-dependent plasticity (STDP)—see, e.g., Markram et al. (2012). For that, the network is exposed to a periodic sequence of external signals, each period corresponding to the one recorded frame, with each frame pixel signal acting on an individual network's neuron. When the frame are applied, each “active” binary pixel is causing the respective neuron to fire. Under the effect of these spikes, the STDP performs either reinforcement or weakening of synaptic weights based on the timing of the spikes of the connected neurons. Practical details of such implementation of the STDP with memristive crossbars are discussed by Prezioso et al. (2016).

For simulation, this method is also the simplest one, giving an explicit expression for each synaptic weight.

#### Quadratic programming

The Hebb rule, described by Equation (3), does not guarantee the perfect recording, because the contributions into *w*_*ij*_, given by each pair of frames, may be (and typically are) mutually contradictory. Better performance may be expected from imposing the minimal requirement for *I*_*i*_^(*q*)^ to have the same sign as the proper next frame's pixel *s*_*i*_^(*q* + 1)^:

(4) $$\begin{array}{r} s_{i}^{\left(q+1\right)}{I_{i}}^{\left(q\right)}\propto \overset{M}{\underset{j\;=\;1}{\sum }}s_{i}^{\left(q+1\right)}s_{j}^{\left(q\right)}w_{ij}>0,\;for\;i=1,2,\ldots N, \end{array}$$

for each pixel of every frame.

According to the algebra basics (see, e.g., p. 13 in Bertsekas, 1995), the system of *NQ* inequalities (4) for 2*N*(*M* – 1) > *NQ* binary weights *w*_*ij*_ only defines a multi-dimensional polygon in the weight space, so that for getting a unique solution for the weight set, it must be complemented with some reasonable additional conditions. With this goal, we have first tried several available algorithms of the linear programming (Vanderbei, 2014); however, they typically lead to growth of the width of the synaptic weight distribution, especially strong at *Q* → *Q*_max_. Such a broad distribution is rather inconvenient for the hardware implementations, in which the range of possible crosspoint device conductances *G* is always limited—see, e.g., Merrikh-Bayat et al. (2015). We have achieved much better results by using the quadratic programming (Best, 2017), in which Equation (4) is complemented with the requirement of the smallest norm of the vector of synaptic weights *w*_*ij*_. The calculations have been performed using the MATLAB's function *quadprog()*.

This recording method is computationally rather intensive, requiring CPU times approximately two orders of magnitude larger than the Hebb rule (3). Also, since the quadratic programming is a global optimization algorithm, we are not aware of any it's possible *in situ* analog-hardware implementation without involving a very significant digital-circuit (i.e., essentially *ex-situ*) overhead.

#### Analog gradient descent

The next natural recoding method is an iterative algorithm similar to the well-known delta-rule of the feedforward perceptron training, describing the gradient descent of the quadratic error function (see, e.g., section 5.4 in Hertz et al., 1991):

(5) $$\begin{array}{r} \Delta w_{ij}=-\eta s_{j}^{\left(q\right)}\varepsilon_{i}^{\left(q+1\right)}. \end{array}$$

Here η is a small training rate (in simulations we used the value η = 0.001, though its choice does not affect the results much), and ε is the error of the previous prediction of the next frame's pixel:

(6) $$\begin{array}{r} \varepsilon_{i}^{\left(q+1\right)}=\overset{M}{\underset{j\;=\;1}{\sum }}w_{ij}s_{j}^{\left(q\right)}-s_{i}^{\left(q+1\right)}. \end{array}$$

Since the weight updates are computed and applied separately for each pair of the consecutive movie frames, this recording algorithm is effectively implementing the stochastic gradient descent optimization with the batch size of one frame pair. The number of times the complete movie is applied to the ASTM represents the number of training epochs.

#### Discrete gradient descent

We have found that the analog gradient descent method may be improved by rounding the sum *a*_*i*_^(*q*)^ to the closest of ±1:

$$\begin{array}{l} \varepsilon_{i}^{\left(q+1\right)}=round\left(a_{i}^{\left(q\right)}\right)-s_{i}^{\left(q+1\right)},a_{i}^{\left(q\right)}=\overset{M}{\underset{j\;=\;1}{\sum }}w_{ij}s_{j}^{\left(q\right)} \end{array}$$

thus limiting the error ε_*i*_^(*q* + 1)^ to the set of values {−2, 0, 2}. However, preliminary testing showed some limitations of this approach, which tends to result in synaptic weights that produce very small values of *a*_*i*_^(*q*)^, and hence unnecessarily increase the system's sensitivity to noise.

This deficiency may be easily eliminated by introducing a small gap with the range [–*D*, +*D*] and modifying the optimization procedure, so that the values of *a*_*i*_^(*q*)^ are “pushed” outside the gap in the process of reducing the cost function. For the case of binary pixels with values ±1, the resulting expression for the error takes form

(7) $$\begin{array}{r} \varepsilon_{i}^{\left(q+1\right)}=S_{i}^{\left(q+1\right)}-s_{i}^{\left(q+1\right)}, \end{array}$$

where the integer *S* depends not only on the current prediction of the output pixel, as in Equation (6), but also on its proper value:

(8) $$\begin{array}{r} S_{i}^{\left(q+1\right)}=\text{sgn}\left(\overset{M}{\underset{j\;=\;1}{\sum }}w_{ij}s_{j}^{\left(q\right)}-Ds_{i}^{\left(q+1\right)}\right). \end{array}$$

Simulations showed that changing the width of the gap *D* (before training) may be used to proportionally scale all the weights of the network, without changing its performance. Therefore, the selection of *D* can be based solely on the implementation convenience. In our simulations we chose *D* = 1. The weights are updated according to Equation (5) with training rate η = 0.005.

### Simulation procedures

The ASTM models discussed in this paper represent complicated nonlinear systems that are difficult to evaluate using analytical methods. Therefore, with one notable exception discussed in section Hebb Rule below, we had to use numerical simulations to estimate the performance characteristics of the system, including first the ASTM capacity, and then its sensitivity to pixel and weight noise.

All simulations have been performed on a square lattice of *N* × *N* neural cells. In order to mitigate the effects of large but finite size *N*, we have used the usual cyclic boundary conditions on both pairs of opposite sides of the square (equivalent to wrapping the network on a thorus).

In order to exclude the effects of hardly-controllable pixel correlation in real-life B/W movies, such as shown in Figure 4, the memories were evaluated on movies composed of fully random frames. This approach may be further justified by taking into account that in order to be recorded in a binary memory like ours, an analog or multi-bit (say gray-scale or color) pixel needs to be represented by many binary pixels, whose correlation, averaged over the whole connectivity domain, is vanishingly small. Besides the data shown in **Figure 9**, the duty cycle of each frame, i.e., the percentage of “active” (say white) pixels was 50%.

Each memory readout trial starts with exposing it to a randomly selected frame of the recorded movie, and then using Equation (1) to sequentially recover all the remaining frames (Since each recorded movie formed a closed loop, the readout continued up until the input frame was reached again). If this last frame virtually matched the input one, the recovery was considered successful. The memory capacity was determined as the maximum length of the movie that could be recorded and read out with a 1% fidelity.

The sensitivity to pixel noise was evaluated by “flipping” a certain percentage of random pixels in the initial frame. In this case, the final frame of each readout attempt was compared with the uncorrupted version of the input one. The sensitivity to pixel noise, was characterized with the percentage of failures to recover the movie correctly, as a function of the corrupted input bit number and the movie length (Typically, the memory either reproduces the movie perfectly after a few first frames, or completely corrupts it).

The sensitivity to weight noise was calculated similarly, except that Gaussian noise was added to each synaptic weight before each readout attempt. In this case, the memory may be capable of reproducing the movie correctly, but with small percentage of wrong pixels in each frame. This is why, in order to evaluate the noise sensitivity, we have set a certain threshold on the percentage of errors in the final frame, used to decide whether the readout is successful. The results presented below are for the thresholds of 1 and 3%.

Due to the stochastic nature of these numerical experiments, getting accurate results requires their averaging over large number of simulation experiments. Each such experiment used for the memory capacity evaluation, included using a new, randomly generated movie, with just one readout run, starting with a random frame (The number of experiments used to obtain each data point is specified in figure captions). The noise sensitivity evaluations were based on 10 series of experiments, with each series using a unique movie and 100 attempts to recover the movie, starting from a random frame, after adding a new random noise pattern—to either the initial frame or to synaptic weights. This procedure provided 1000 data points for each final (average) point shown below.

## Results

### Hebb rule

Since the Hebb Rule gives an explicit expression (3) for the synaptic weights, the resulting capacity-to-fidelity tradeoff may be readily evaluated analytically, assuming that all the binary pixels in the whole movie are random and uncorrelated. Indeed, let us assume that in a frame number *q*, all *M* cells within the connectivity domain of an *i*th cell have correct values: *V*_*j*_^(*q*)^ = *V*_0_*s*_*j*_^(*q*)^. Then plugging Equation (3) (with the summation index replacement *q*→*q'*) into Equation (1), we may calculate the normalized product of the signal *I*_*j*_ arriving at the *j*^th^ cell, by the sign of its correct value, *s*_*i*_^(*q* + 1)^, in the next frame:

(9) $$\begin{array}{r} \frac{Q}{V_{0}}I_{i}^{\left(q\right)}s_{i}^{\left(q+1\right)}=\overset{M}{\underset{j\;=\;1}{\sum }}\overset{Q}{\underset{q^{\prime }\;=\;1}{\sum }}s_{i}^{\left(q+1\right)}s_{i}^{\left(q^{\prime }+1\right)}s_{j}^{\left(q^{\prime }\right)}s_{j}^{\left(q\right)}. \end{array}$$

Due to the independence of different pixels, the sum of *MQ* terms in the right-hand part of Equation (9) has only *M* terms (all with *q* = *q'*) always equal to +1, while all other terms have an equal probability to equal either +1 or−1. At *M, Q* >> 1, the sum of these *M*(*Q* – 1) random terms has a Gaussian probability distribution with a zero statistical average, and the variance equal to *M*(*Q* – 1) ≈ *MQ*. As a result, the probability of the negative sign of the whole sum (Equation 9), i.e., of an error of the *i*th pixel in the (*q* + 1)^st^ frame, is

(10) $$\begin{array}{r} p\approx \frac{1}{\sqrt{2\pi MQ}}\int_{M}^{\infty }\exp\left(-\frac{x^{2}}{2MQ}\right)dx\equiv \frac{1}{2}\text{erfc}\sqrt{\frac{M}{2Q}}, \end{array}$$

where erfc(*x*) = 1 – erf(*x*) is the complementary error function. Note that this result is similar to that for the Hopfield networks with the similarly sharp activation function (see, e.g., section 2.2 in Hertz et al. (1991), and similarly restricted connectivity (Türel et al., 2004), because for this calculation, the addition of 1 to the upper indices in the right-hand part of Equation (9) is not important.

In the most important limit of small error probability, Equation (10) is reduced to

(11) $$\begin{array}{r} p\approx \sqrt{\frac{Q}{2\pi M}}\exp\left\{-\frac{M}{2Q}\right\}<<1,\text{ for }1<<Q<<M \end{array}$$

**Figure 5:**
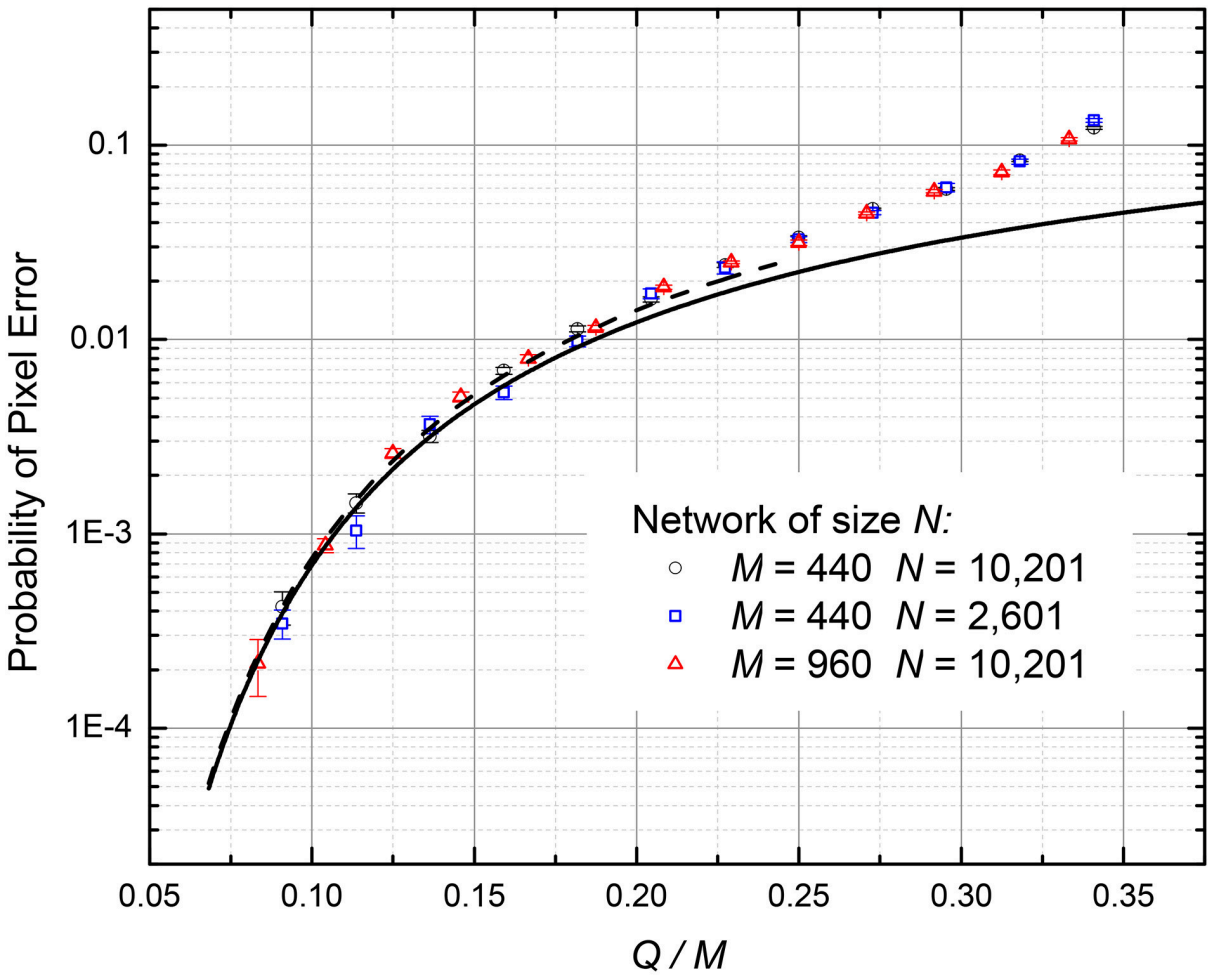
The probability of pixel retrieval error in the ASTM using the Hebbian recording (Equation 3), as a function of the normalized number *Q* of the recorded frames. Lower curve: Equation (10). Dashed curve: Equation (11), valid only for small probability of pixel errors. Upper points: numerical simulation results, which automatically take into account the induced errors.

At larger *p*, we need to take into account the induced errors, i.e., the effect of an error in a *q*^th^ frame on the error probability in the (*q* + 1)^st^ frame. At *Q, M* >> 1, such a calculation may be performed analytically using the mean-field approach, similar to that used for the calculation of the Hopfield network's capacity—see, e.g., section 2.5 in Hertz et al. (1991). However, since the main focus of this work was on other recording methods, giving better results (see below) we have opted for the simple numerical simulation of the readout. These numerical experiments have shown that at the retrieval process, the fraction of incorrect pixels per frame rapidly approaches some stationary, equilibrium value *p*; these values are plotted by points in Figure 5 for several *N* and *M*. For all the simulated cases, the normalized memory capacity *Q*_max_/*M* is virtually independent of these parameters—just as in Equations (10) and (11).

For the practically interesting fidelity range (*p* ≤ 1%), the corrections due to induced errors are not important, and the numerical results, with a good accuracy, are described by Equations (10) and (11). In particular, for the 99% fidelity (*p* = 0.01), *Q*_max_ ≈ 0.18*M*. Such low capacity is not too surprising, given the well-known result *Q*_max_ ≈ 0.14*M* for the Hopfield networks with the similarly restricted connectivity (Türel et al., 2004), and the similar activation function.

### Quadratic programming

The simulations have shown that with the growth of the number *Q* of the recorded frames, the correct retrieval degradation is different from that at the Hebb-rule recording. Namely, the number of wrong pixels in each retrieved frame is typically very small, but when a few errors appear, they almost immediately lead to a complete corruption of the remaining frames of the movie. As a result, the system's fidelity violation is better characterized by the probability *p* of the movie corruption, measured on a large statistical ensemble of different movies (again, with completely random and independent pixels).

**Figure 6:**
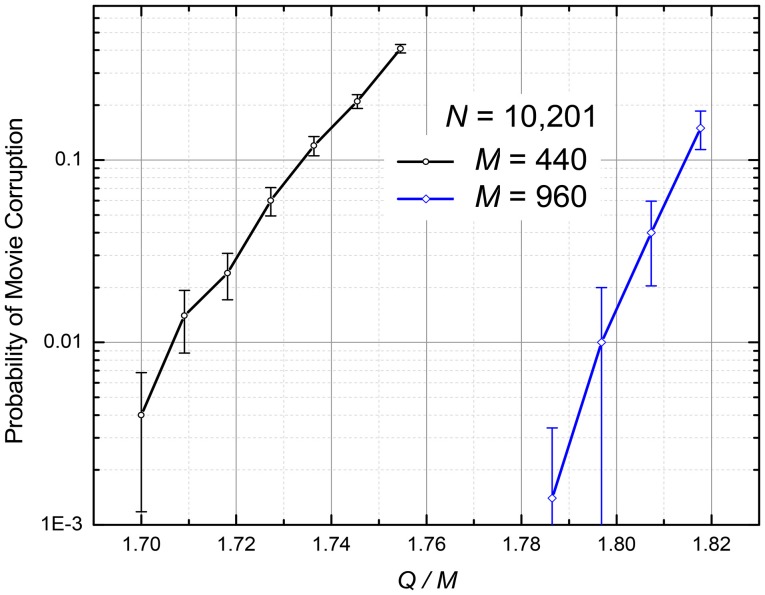
The numerically calculated probability of the retrieved movie's corruption at the quadratic programming (The curves are only guides for the eye). The error bars represent standard deviation of the mean based on 500 simulations for *M* = 440 and 100 simulations for *M* = 960.

Figure 6 shows the *p* so defined as a function of the same ratio *Q*/*M* as in Figure 5. The results, which were obtained by first recording and then replaying randomly generated movie for each simulation run, show that for a reasonable fidelity (say, *p* = 1%), the network capacity *Q*_max_, averaged over two simulated cases (*M* = 440 and *M* = 960), is (1.75 ± 0.05)*M*, i.e., is almost an order of magnitude higher than for the Hebb-rule recording.

Note that for the case of global connectivity (*M* = *N* – 1), this number is close to the theoretical capacity maximum *Q*_max_ = 2(*N* – 1) of the usual (spatial) associative memory, based on a recurrent neuromorphic network (Gardner and Derrida, 1988).

### Analog gradient descent

The data recording was performed by iteratively updating weights according to Equations (5) and (6) until the optimization algorithm converged to global minimum of prediction error. We assumed that the solution is near the global minimum when the magnitudes of all errors (Equation 6) drop below 0.1. Preliminary testing showed that setting a more stringent criterion did not improve the capacity or noise sensitivity of the network. In cases when the algorithm was not converging, iterations were stopped after 10^5^ epochs.

**Figure 7:**
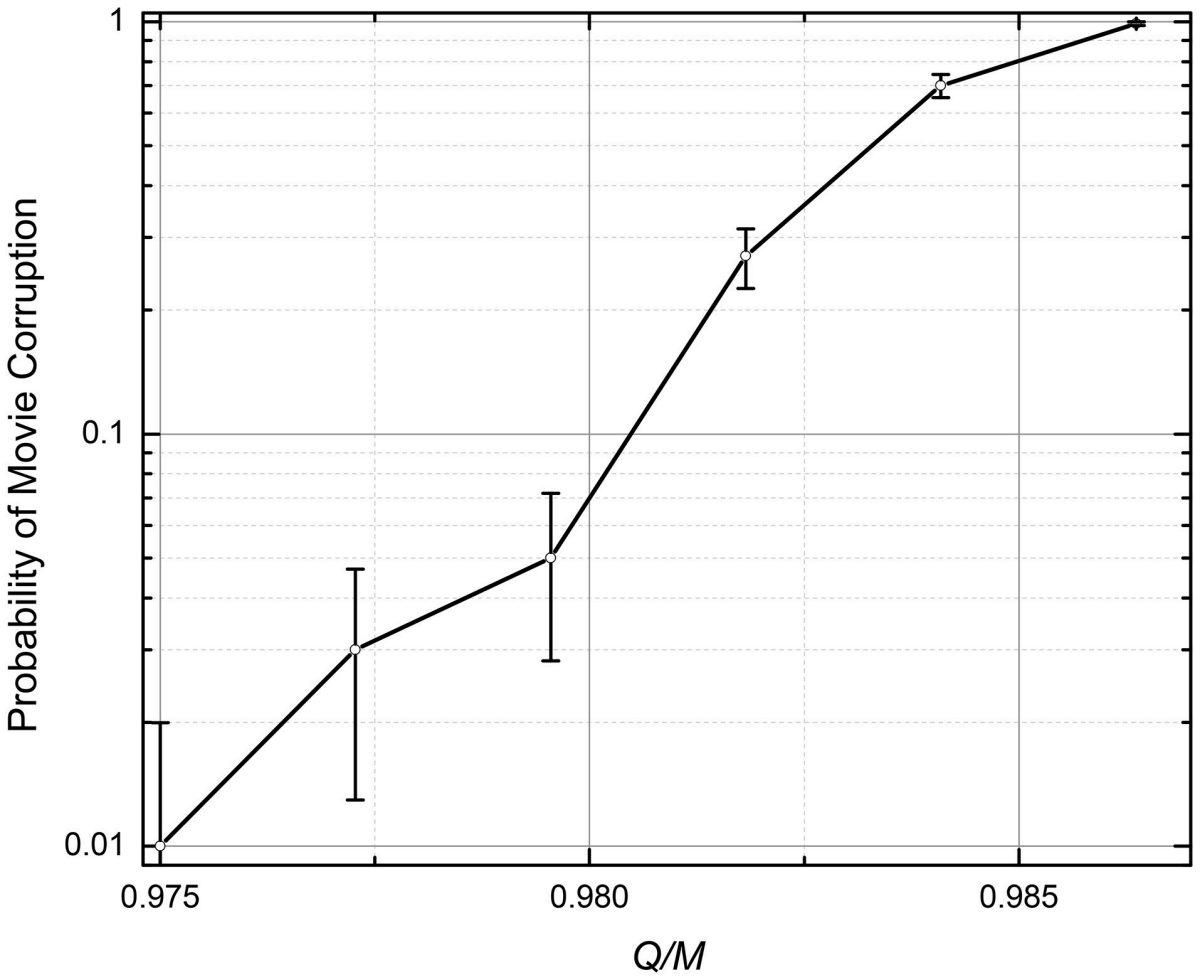
The probability of the retrieved movie's corruption at the analog gradient-descent recording (The lines are only guides for the eye). *N* = 101 × 101 = 10,201, *M* = 21 × 21 – 1 = 440, η = 10^−3^. The recording iterations (Equation 5) were stopped either after 10^5^ epochs, or when the magnitude of all errors (Equation 6) dropped below 0.1. The error bars represent the standard deviation of the mean based on 100 simulations.

**Figure 8:**
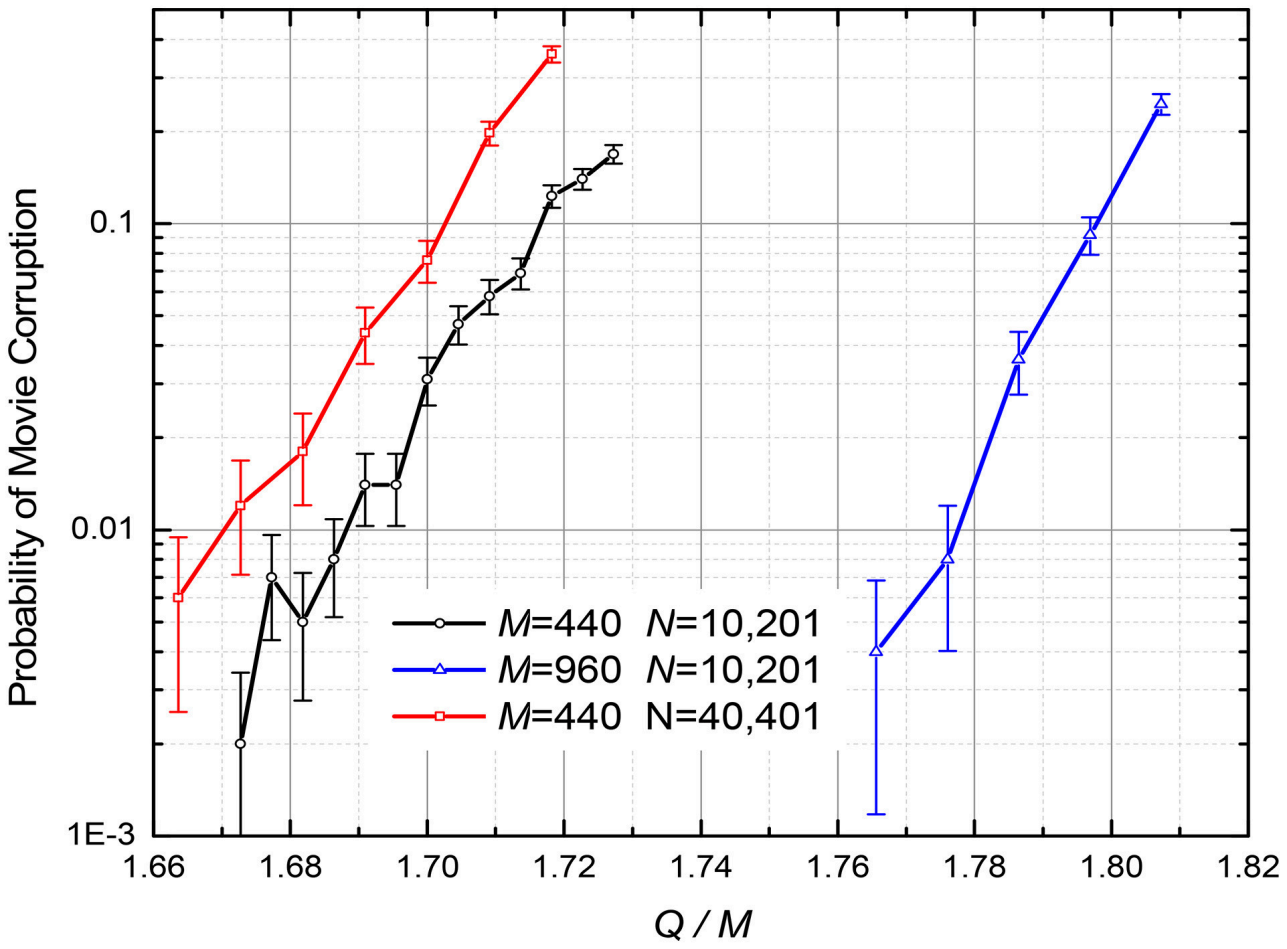
The probability of the retrieved movie's corruption at the discrete gradient-descent recording described by Equations (5), (7), and (8) (The lines are only guides for the eye). η = 0.01; *D* = 1. The recording iterations were stopped when the errors ε_*i*_^(*q* + 1)^, defined by Equation (7), reached 0 for all *i* and *q*. The error bars represent the standard deviation of the mean, based on 1,000 simulations for *M* = 440 and 500 simulations for *M* = 960.

The numerical simulation has shown that the movie retrieval dynamics is qualitatively similar to that for the quadratic programming (see the previous subsection): an increase of the number *Q* of the recorded frames leads to an increase of the probability *p* of the total corruption of the retrieved movie. Figure 7 shows a typical dependence of this probability on the ratio *Q*/*M*; it indicates that the memory's capacity is approximately two times lower than that for the quadratic-programming recording; for *p* = 1%, *Q* ≈ 0.97*M* [Similarly to the shown QP results, in Figure 7 (and Figure 8 below) each simulation run involved recording and replaying randomly generated movie].

In hindsight, such relatively poor results might be anticipated. Indeed, the algorithm (Equations 5, 6) forces the network outputs to approach the *exact* integer values *s*_*i*_^(*q* + 1)^ of the next pixels, while for the successful movie retrieval, it is only necessary for it to have its sign correct—see Equation (2). As the result, the unnecessary changes of the weights interfere with the substantial ones, and hinder the iterations' efficiency.

### Digital gradient descent

The ASTM recording procedure was organized similarly to that for Analog Gradient Descent, except that in the iterative optimization algorithm weight updates are determined by Equations (5), (7), and (8). Figure 8 shows the probability of the retrieved movie corruption as a function of the normalized number *Q* of the recorded frames, for several values of parameters *N* and *M*. The results imply that the capacity-to-fidelity tradeoff is almost as good as that available from the (much less convenient) quadratic programming; for example, at *p* = 1%, *Q*_max_ ≈ (1.67 ± 0.02) *M*, depending on *M* and *N*.

**Figure 9:**
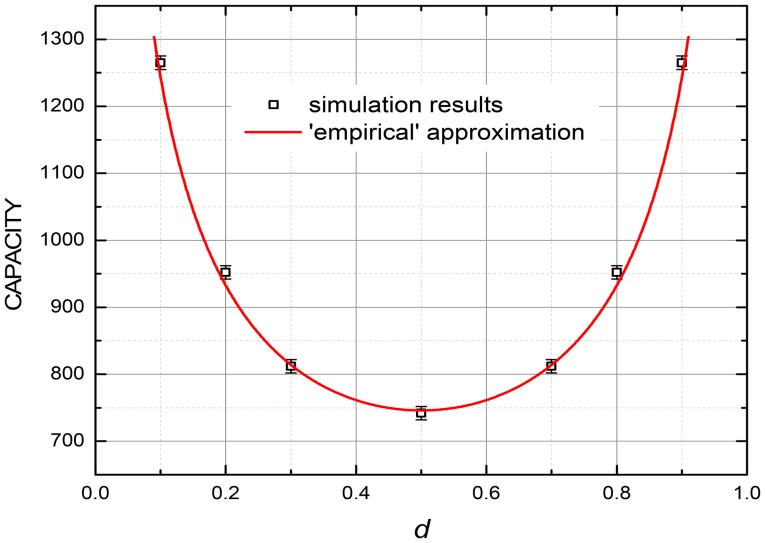
The capacity (at 99% fidelity) at the discrete gradient-descent recording, as a function of the “duty cycle” *d*, i.e., the fraction of binary pixels having a certain binary value in each frame, for *M* = 440. The smooth curve shows the empirical dependence *Q*_max_ ∞ 1/[*d*(1 – *d*)]^1/2^. The error bars represent the estimated maximum deviation.

Note that all the CrossNet ASTM capacity results, shown in Figures 5–8, are for completely random binary (B/W) pixels, i.e., for the 50% probability for each pixel to have a certain value (±1). If this probability is either lower or higher, the capacity is even larger—see, e.g., the results shown in Figure 9.

For very sparse patterns (with either *d* << 1 or 1 – *d* << 1), even higher capacity may be possible using a natural modification of the recording rules suggested for usual (spatial) associative memories—see, e.g., pp. 52–53 in Hertz et al. (1991). At the software implementations of the memories, these rules are sometimes applied to dense patterns (with *d* ~ ½) as well, using their mapping on sparse ones. At the hardware implementation, however, such approach would require an impracticable increase of the necessary resources.

### Immunity to noise and device variability

To summarize the previous section, two of the methods we have studied, stand out of the competition: the first method, based on the quadratic programming, due to the largest memory capacity, and the second one (based on a discrete version of the gradient descent approach) due to its local nature, enabling hardware implementation of the recording, with a minimal involvement of peripheral circuitry—at a very competitive capacity. These two methods have been chosen for a more detailed study, namely a numerical evaluation of the CrossBar ASTM's immunity to the noise contamination of the input frame, and of its tolerance to random deviations of the synaptic weights from the optimal values calculated at the recording (Such deviations are currently the largest challenge for large-scale applications of metal-oxide memristors and other species of these prospective devices, fabricated using various technologies). Random deviations of weights were simulated by adding random deviations to the original weights before each movie retrieval attempt. The deviations were random and independent, obeying the Gaussian distributions with zero mean, and a relative r.m.s. value *r*.

**Figure 10:**
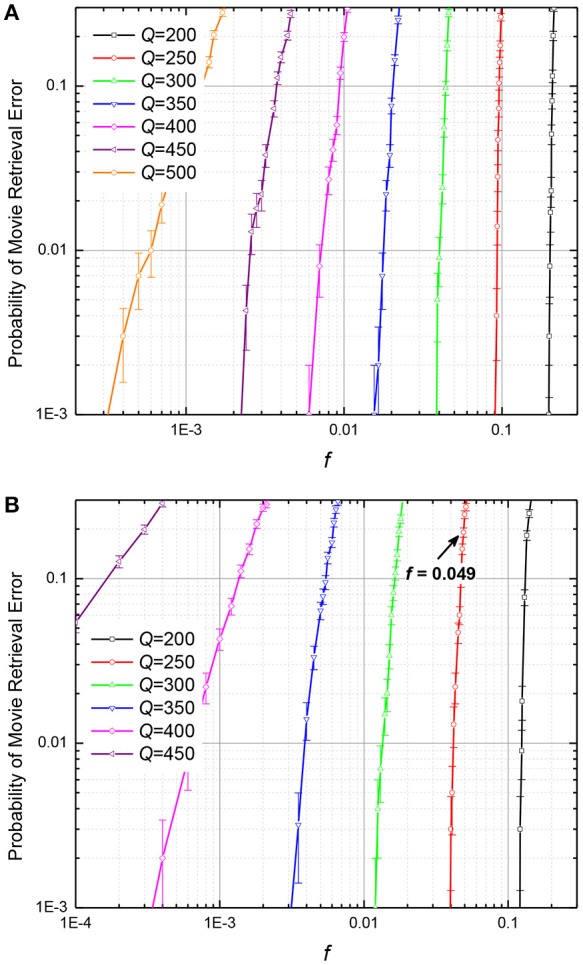
The probability of movie retrieval error at: **(A)** the quadratic-programming recording, and **(B)** the discrete gradient-descent recording, as functions of the fraction *f* of wrong (randomly flipped) binary pixels in the input frame, for *M* = 440. The arrow shows the point with *f* = 0.049, which corresponds to the case illustrated in Figure 12. The error bars represent standard deviation of the mean based on 1,000 simulations (10 randomly generated movies, 100 tests per movie).

**Figure 11:**
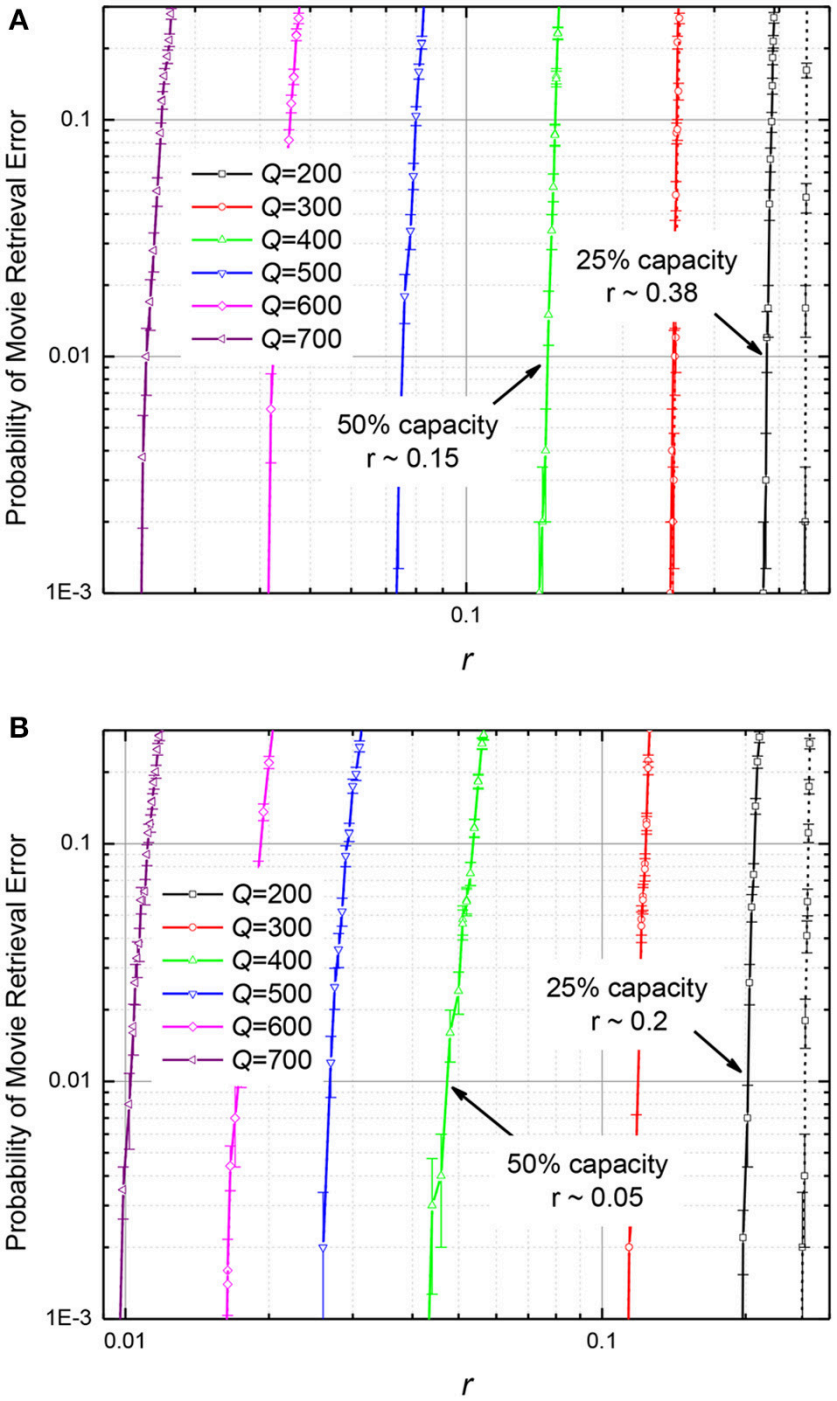
The probability of movie retrieval error in the ASTMs with *M* = 440, using **(A)** the quadratic-programming recording, and **(B)** the discrete gradient-descent recording, as functions of the normalized (relative) r.m.s. deviation *r* of the synaptic weights from the optimal (calculated) values. Solid lines represent the 99% fidelity of recovery, while the dashed lines, the 97% fidelity. The error bars represent standard deviation of the mean based on 1000 simulations (10 randomly generated movies, 100 tests per movie).

The results of these calculations are presented, respectively, in Figures 10, 11. The plots in Figure 10 show, for example, that if the number *Q* of frames recorded into an ASTM, by either of the two methods, is at 25% of its maximum capacity (*Q* = 200), it may recognize the input frame with ~10% corrupted pixels, but if *Q* is increased to 400, i.e., to 50% of *Q*_max_, the input noise tolerance drops sharply, to only ~10^−3^ of the pixels.

On the contrary, as Figure 11 shows, filling of memory has smaller effect on its tolerance to fluctuations of memristive device conductance. For example, if the ASTM with the quadratic programming recording (Figure 11A) is filled to 25% of its maximal capacity, its operation is not hindered by weight fluctuations with ~38% relative r.m.s. If additional data is written to such memory, so that it is filled to 50% of capacity, weight fluctuations with the r.m.s. above ~15% cause movie corruption. For the discrete gradient descent recording (Figure 11B), the fluctuation tolerance is ~20% for the 25% memory fill, and ~5% for the 50% memory fill.

**Figure 12:**
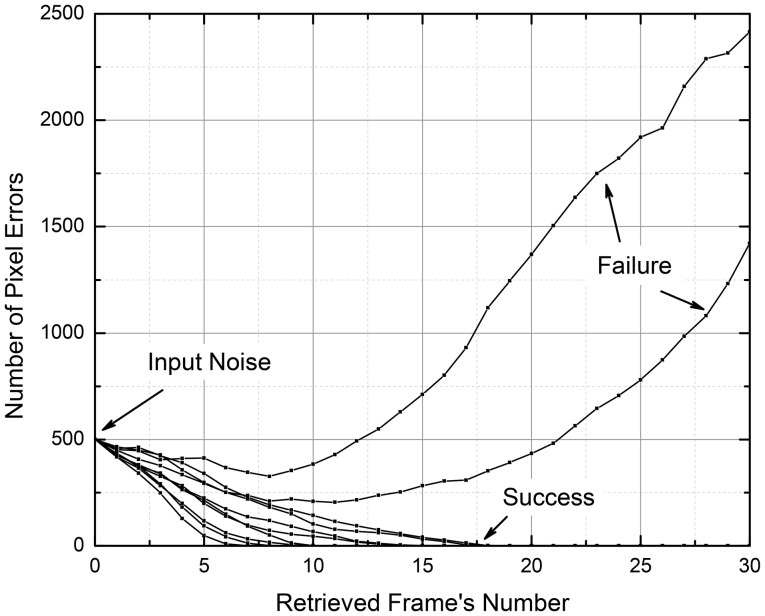
The input pixel noise suppression by a CrossNet with *N* = 101 × 101 = 10,201; *M* = 21 × 21 – 1 = 440 in the process of movie retrieval, as simulated for 10 independent random noise patterns. The recording of the movie with *Q* = 250 frames was performed using the discrete gradient descent method.

It is important to note that these results characterize not an instant, but rather a gradual suppression or amplification of the input noise. For example, Figure 12 shows the number of wrong pixels in *N* = 10,201-pixel frames for 10 simulated movie retrievals, for a system with the cell connectivity *M* = 440, with *Q* = 250 frames recorded using the discrete gradient descent method. The plots show that all 500 input errors (which were independent for each retrieval attempt) eventually disappeared in 8 cases, but led to a full movie corruption in two cases (These data are a small part of a set of 1,000 movies, which gave the point with *f* = 500/10,201 ≈ 0.049 and the error probability ~0.2—see the point marked in Figure 10B).

### Comparison with T-CAM

**Figure 13:**
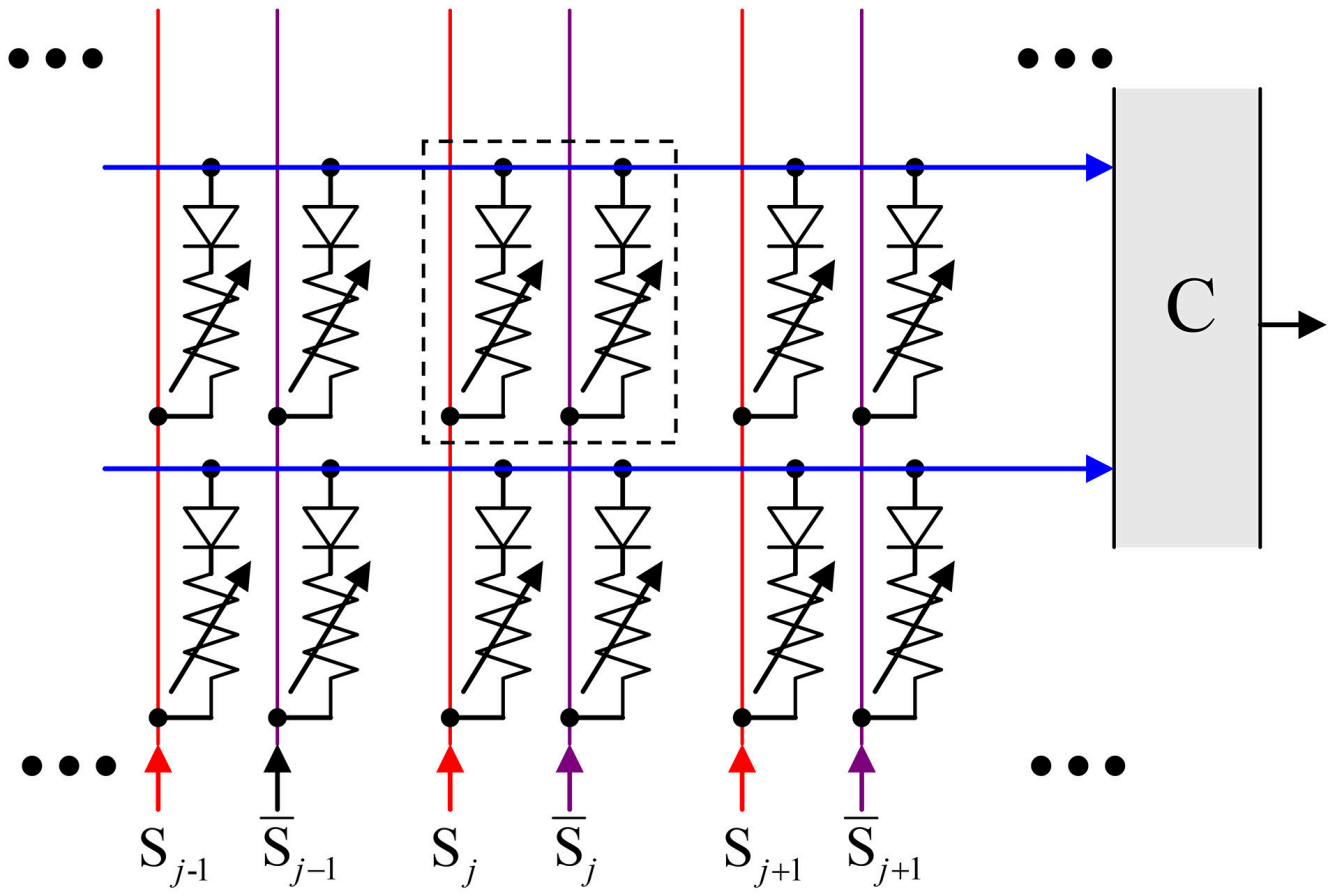
An ASTM implemented as a memristive Ternary Content-Addressable Memory (T-CAM).

The results shown in Figures 10, 11 need to be compared with those for the main competitor of the CrossNet ASTM, the T-CAM circuits already mentioned in the Introduction. Figure 13 shows a 2 × 3-cell fragment of the memristive T-CAM (Alibart et al., 2011). It is a rectangular matrix of cells, with two binary-state crosspoint devices (plus two diodes) per cell, with each bit stored in the complimentary binary states (ON and OFF) of these two devices, whose order encodes the bit.

Using the same movie language, the *N* binary pixels of each frame are stored in one row of such ASTM, so that the storage of *Q* frames requires *Q* rows. Before the movie retrieval, the row lines are pre-charged to the same voltage *V*_0_. The retrieval is induced by feeding each pair of column lines with voltages {*V*_0_, 0}, in the order dictated by the value of the corresponding binary pixel of the input frame. If the bit recorded in a cell corresponds to the input bit (i.e., if the input voltage *V*_0_ > 0 corresponds to the ON state of the corresponding crosspoint device, with a high conductance, while the input voltage 0, to the OFF state, with its very low conductance), the feed does not result in a noticeable current through the cell. As a result, if a recorded frame exactly matches the input one, the row line's voltage stays high. On the contrary, if some bits of a recorded frame are different from those of the input frame, the corresponding row line discharges, with the rate proportional to the number of misfit bits, i.e., to the Hamming distance between these two-bit strings. The discharge rates of all rows are compared by the comparator C, and the row with the slowest rate is assumed to carry the requested frame. After the choice of the row has been made, the whole movie may be played out without any further input (This design may be readily generalized to more than two dimensions—see, e.g., Imani et al., 2017).

The fact that this circuit requires *n* = 2*NQ* memristive devices (besides the diodes and the peripheral circuits including the multi-input comparator) may be represented by saying that the frame capacity of the T-CAM with *n* devices is

(12) $$\begin{array}{r} Q_{\max}=\frac{n}{2N}. \end{array}$$

This value should be compared with the result *Q*_max_ ≈ 1.75*M* = (7/8)*M* at 1% fidelity for the CrossNet ASMT discussed in this paper (for the two best recording methods). Since in that memory, with the differential encoding of the synaptic weights, the total number of crosspoint devices is *n* = 2*MN*, that result may be rewritten as

(13) $$\begin{array}{r} Q_{\max}\approx \frac{7}{8}\frac{n}{N}, \end{array}$$

i.e., the capacity (Equation 12) of the T-CAM with the same number of devices is a factor of 7/4 lower.

If the frame of *N* binary pixels, submitted to T-CAD for the recognition, has some number (say, *fN*) of corrupted pixels, there is a chance that its Hamming distance from a wrong recorded frame will be lower than that from the correct frame, so that the memory will recall that wrong frame. Since the Hamming distance between two random strings, of *N* >> 1 bits each, obeys the Gaussian distribution with the mean *N*/2 and the variance *N*/4, the probability of such an error is

(14) $$\begin{array}{l} p=\frac{1}{\sqrt{2\pi \left(N/4\right)}}\overset{fN}{\underset{0}{\int }}\exp\left[-\frac{{\left(k-N/2\right)}^{2}}{2\left(N/4\right)}\right]dk\\ \equiv \frac{1}{2}\left\{\text{erf}\left[\left(2f-1\right)\sqrt{\frac{N}{2}}\right]-\text{erf}\left(-\sqrt{\frac{N}{2}}\right)\right\}. \end{array}$$

According to this formula, at *N* >> 1 the error is extremely small until the fraction *f* of the pixels in the input frame approaches 50% very closely—by the distance of the order of 1/(2*N*)^1/2^ << 1. Hence, the noise immunity of the T-CAM is higher than that of the CrossNet ASMT—cf. Figure 10.

The crosspoint device fluctuation tolerance of the T-CAM is also higher than that in the CrossNet ASTM. In order to characterize it, we should take into account that the Ohmic conductance *G* of real-life memristors is non-vanishing even in the OFF state. Hence the voltage decay rate in the line corresponding to the perfect fit to the input frame (Figure 13) is *NV*_0_*G*_OFF_ > 0. On the other hand, the average rate of a misfit line discharge is *NV*_0_*G*_ON_/2, with an r.m.s. fluctuation scaling as √*N* << *N*. Hence an error due to the worst-case (simultaneous) fluctuations of the conductances appears only at

(15) $$\begin{array}{r} {\left(G_{\text{OFF}}\right)}_{\max}>\frac{1}{2}{\left(G_{\text{ON}}\right)}_{\max} \end{array}$$

- the situation at which a memristive array is typically considered even unworthy of testing.

## Conclusion

Our calculations have shown that hybrid CMOS/memristor circuits with the CrossNet architecture may be indeed used as ASTM, especially if operated in the synchronous mode, with the global external timing of all neural cells. Of the studied information recording methods, two gave the best results for the capacity-fidelity tradeoff and noise tolerance: one using the quadratic programming approach, the second one based on a discrete version of the gradient descent method (The latter method, while providing a slightly lower capacity, is more convenient for the hardware-based recording).

With any of these recording methods, the CrossNet ASTMs may be more hardware-saving than the alternative, T-CAM circuits of the same capacity, by offering higher data recording density per memristor, though the input noise immunity and memristor variability tolerance of the CrossNet ASTM are lower. It is important to note that CrossNet ASTM's capacity increases naturally, without any modifications to the network, for more realistic cases of correlated frames (see Figure 9). On the other hand, T-CAM implementations would have to rely on coding and/or compression algorithms, which might have substantial implementation overhead and inferior information capacity.

One more challenge for the experimental implementation of the CrossNet ASCM is the still immature technology of memristor hybridization with underlying CMOS circuits (Chakrabarti et al., 2017). However, the field of possible applications of our results is much broader than the memristor-based networks. For example, they are fully applicable to CrossNet-like circuits using floating-gate memory cells, with analog data recording, as synapses—see, e.g., Hasler and Marr (2013). At the industrial-grade implementation of such cells, they may be quite comparable with memristors in size, and provide almost similar speed and energy efficiency. The recent fast progress of experimental work in this direction (Guo et al., 2016; Merrikh Bayat et al., in press) gives every hope that the CrossNet ASTMs based on such technology may become valuable components of future ultrafast cognitive hardware systems.

## Author contributions

DG performed numerical studies of ASCM. KL and DS performed the theoretical analysis and supervised the project. All coauthors participated in numerous discussions of the results.

### Conflict of interest statement

The authors declare that the research was conducted in the absence of any commercial or financial relationships that could be construed as a potential conflict of interest.
